# Integrating pharmaceutical systems strengthening in the current global health scenario: three ‘uncomfortable truths’

**DOI:** 10.1186/s40545-020-00242-2

**Published:** 2020-06-25

**Authors:** Tamara Hafner, Marlon Banda, Jillian Kohler, Zaheer-Ud-Din Babar, Murray Lumpkin, Mojisola Christianah Adeyeye, Emmanuel Nfor, Francis Aboagye-Nyame, Javier Guzman

**Affiliations:** 1 USAID Medicines, Technologies, and Pharmaceutical Services (MTaPS) Program, Management Sciences for Health, Arlington, VA USA; 2 Churches Health Association of Zambia, Lusaka, Zambia; 3 Leslie Dan Faculty of Pharmacy & Dalla Lana School of Public Health & Munk School of Global Affairs & Public Policy, University of Toronto, Toronto, ON Canada; 4 Department of Pharmacy, University of Huddersfield, Queensgate, Huddersfield HD1 3DH UK; 5 Bill and Melinda Gates Foundation, Seattle, WA USA; 6 National Agency for Food and Drug Administration and Control, Abuja, Nigeria

**Keywords:** Pharmaceutical systems strengthening, Access to medicines

## Abstract

The response to emergency public health challenges such as HIV, TB, and malaria has been successful in mobilising resources and scaling up treatment for communicable diseases. However, many of the remaining challenges in improving access to and appropriate use of medicines and services require pharmaceutical systems strengthening. Incorporating pharmaceutical systems strengthening into global health programmes requires recognition of a few ‘truths’. Systems strengthening is a lengthy and resource-intensive process that requires sustained engagement, which may not align with the short time frame for achieving targets in vertical-oriented programmes. Further, there is a lack of clarity on what key metrics associated with population and patient level outcomes should be tracked for systems strengthening interventions. This can hinder advocacy and communication with decision makers regarding health systems investments. Moving forward, it is important to find ways to balance the inherent tensions between the short-term focus on the efficiency of vertical programmes and broader, longer-term health and development objectives. Global health programme design should also shift away from a narrow view of medicines primarily as an input commodity to a more comprehensive view that recognizes the various structures and processes and their interactions within the broader health system that help ensure access to and appropriate use of medicines and related services.

## Introduction

Low- and middle-income countries (LMICs) are undergoing a triple transition that involves a shift from development assistance to domestic resources, an epidemiological transition from communicable to noncommunicable diseases as the major source of morbidity and mortality, and a reorganisation of national health systems to meet universal health coverage (UHC) goals as expressed in Target 3.8 of the Sustainable Development Goals (SDGs) [1]. Noncommunicable diseases such as cancer, strokes, chronic respiratory infections, hypertension, and diabetes are responsible for more than 32 million deaths per year in LMICs [2]. Global health programmes and partnerships such as the Global Fund and The President’s Emergency Plan for AIDS Relief (PEPFAR) have been tremendously successful in mobilising resources and scaling up treatment for communicable diseases [3–6]. However, these initiatives are unsustainable in the absence of donor support and insufficient for tackling the challenges that come with the triple transition. The need to respond to emergency public health challenges such as HIV, TB, and malaria has led to an undue emphasis on the supply chains that deliver commodities required for the diagnosis and treatment of these diseases.

Many of the challenges that remain in ensuring a population’s access to and appropriate use of medicines and services, particularly in the context of the health-related SDGs, require pharmaceutical systems strengthening approaches. This goes beyond the narrow focus of avoiding stockouts of a subset of health products. Pharmaceutical systems strengthening recognises the various structures, processes, and their interactions within the broader health system that help to ensure access to and appropriate use of safe, effective, quality medicines [7]. However, acknowledging the centrality of pharmaceutical systems strengthening and incorporating it into the current global health scenario require recognition of a few interrelated and 'uncomfortable truths'.

## Three truths

First, pharmaceutical systems strengthening is a lengthy and resource-intensive process that may not align with the short time frame for achieving targets in donor-funded programmes. For example, medicines and medical device registration is normally performed by a national regulatory agency before products can be used in the country. Many national regulatory agencies, especially in LMICs, face significant challenges, including inadequate funding, human resources, regulatory capacity and subject matter expertise, the fragmentation of regulatory functions, critical human resource shortages, and lack of sustainable funding. As a result of this systemic weakness, medicines registration decisions in many LMICs can be unnecessarily prolonged and burdensome. Global health programmes usually rely on stringent regulatory authorities or the World Health Organization (WHO) prequalification programme to assure the quality of the products they are purchasing. This approach, along with special WHO programmes to expedite national registration based on WHO prequalification, successfully addresses the short-term goal of making products procured by global health programmes quickly available to address urgent needs [8]. However, strengthening national regulatory capacity to ensure that all medical products circulating in a country are safe, effective, and quality assured requires a much longer time commitment and more resources.

Second, pharmaceutical systems strengthening includes strengthening governance through interventions such as the reform of policies and legislation; creation of organisational structures for appropriate decision making, authority, and oversight; and incorporating tenets of good governance (such as transparency, accountability and civil society participation) in systems and processes [9]. Such interventions require country ownership and sustained engagement with multiple stakeholders, some of whom are beyond the immediate health sector. For example, sustained engagement with multiple stakeholders including the ministries of health, justice and agriculture, private sector pharmacy groups and universities has proven critical for building political will and maintaining momentum during the protracted legislative reform needed in instances such as providing the necessary legal mandate for establishing a medicines regulatory authority or requiring the use of the national essential medicines list as the sole list for public procurement [9]. And yet, attempts to integrate disease-specific programmes into national health systems often have insufficient effort focused on national ownership, stewardship, and other health system functions [10–12]. Further, to the extent that other health system functions are considered, their importance is often limited to how well they facilitate the achievement of specific programme targets.

Third, it is not clear what systems strengthening interventions work and why. A variety of tools exist to assess health systems [13–15], and recent efforts have sought to document the outcomes and impacts of systems strengthening interventions on health [16–18]. However, there is a lack of clarity on what key metrics associated with population and patient-level outcomes should be tracked to evaluate pharmaceutical systems strengthening interventions. On the other hand, disease-specific programmes have been tracking population outcomes to allow for effective communication and advocacy and resonance with decision makers. We know, for example, that the Global Fund has saved 32 million lives as of the end of 2018, and that overall, the number of deaths caused by AIDS, TB, and malaria each year has decreased by 40% since 2002 in countries where the Global Fund invests [4]. The lack of consensus on reliable measures for evaluating pharmaceutical systems strengthening efforts limits the ability to make persuasive investment cases for national governments and donors, who need robust evidence for decision-making and optimizing the allocation of limited resources. The WHO Global Benchmarking Tool for evaluation of national regulatory systems (GBT) and the WHO Joint External Evaluation tool are two globally accepted tools that could serve as models for a possible approach to measurement and incentivizing countries to assess their pharmaceutical systems. WHO recently finalised the GBT, which is the first internationally agreed-upon tool for objectively assessing the functionality and capacities of national medicines regulatory systems and creating an institutional development plan for countries. Similarly, the WHO Joint External Evaluation tool assesses countries’ ability to prepare for and respond to infectious disease risks and prioritizes areas for improvement. The multisectoral approaches of the WHO Joint External Evaluations and heavy reliance on multi-stakeholder engagement could also provide lessons for sustaining stakeholder engagement and multisectoral coordination, which is critical given the multisectoral approaches required for the SDGs.

## Conclusion

Public health challenges such as HIV and TB, and more recently emerging threats such as COVID-19 have underscored the need for resilient health systems in mounting an effective public health response. Moving forward, it is important to find ways to balance the inherent tensions between the short-term focus on the efficiency of disease-specific programmes and broader, longer-term health and development objectives that require high-performing, sustainable health systems. This will also require a shift away from viewing medicines solely as an input commodity, or supply chain management concern, to a more holistic and comprehensive view that recognizes the various structures, processes and their interactions within the broader health system that help ensure access to and appropriate use of medicines and related services. It is also important to build the evidence base and agree on the metrics for evaluating the effectiveness of pharmaceutical systems strengthening interventions with respect to health and population outcomes. This requires implementation research to identify effective interventions and better understand what approaches work and why, which can then inform policy advocacy and action. Globally agreed-upon measures and benchmarks could also provide incentives for standardising country-level measures and for countries to use them to monitor and strengthen their systems.

## Data Availability

Not applicable.

## Abbreviations

GBT: WHO Global Benchmarking Tool for evaluation of national regulatory systems
LMICs: Low- and middle-income countries
PEPFAR: The President’s Emergency Plan for AIDS Relief
SDG: Sustainable Development Goals
UHC: Universal health coverage
WHO: World Health Organization
